# Effects of varying Notch1 signal strength on embryogenesis and vasculogenesis in compound mutant heterozygotes

**DOI:** 10.1186/1471-213X-10-36

**Published:** 2010-03-29

**Authors:** Changhui Ge, Pamela Stanley

**Affiliations:** 1 Department of Cell Biology, Albert Einstein College of Medicine, New York, NY-10461, USA; 2 Beijing Institute of Radiation Medicine, Beijing, 100850, PR China

## Abstract

**Background:**

Identifying developmental processes regulated by Notch1 can be addressed in part by characterizing mice with graded levels of Notch1 signaling strength. Here we examine development in embryos expressing various combinations of *Notch1 *mutant alleles. Mice homozygous for the hypomorphic *Notch1*^12*f *^allele, which removes the single *O*-fucose glycan in epidermal growth factor-like repeat 12 (EGF12) of the Notch1 ligand binding domain (lbd), exhibit reduced growth after weaning and defective T cell development. Mice homozygous for the inactive *Notch1*^*lbd *^allele express Notch1 missing an ~20 kDa internal segment including the canonical Notch1 ligand binding domain, and die at embryonic day ~E9.5. The embryonic and vascular phenotypes of compound heterozygous *Notch1*^12*f*/*lbd *^embryos were compared with *Notch1*^+/12*f*^, *Notch1*^12*f*/12*f*^, and *Notch1*^*lbd*/*lbd *^embryos. Embryonic stem (ES) cells derived from these embryos were also examined in Notch signaling assays. While Notch1 signaling was stronger in *Notch1*^12*f*/*lbd *^compound heterozygotes compared to *Notch1*^*lbd*/*lbd *^embryos and ES cells, Notch1 signaling was even stronger in embryos carrying *Notch1*^12*f *^and a null *Notch1 *allele.

**Results:**

Mouse embryos expressing the hypomorphic *Notch1*^12*f *^allele, in combination with the inactive *Notch1*^*lbd *^allele which lacks the Notch1 ligand binding domain, died at ~E11.5-12.5. *Notch1*^12*f*/*lbd *^ES cells signaled less well than *Notch1*^12*f*/12*f *^ES cells but more strongly than *Notch1*^*lbd*/*lbd *^ES cells. However, vascular defects in *Notch1*^12*f*/*lbd *^yolk sac were severe and similar to *Notch1*^*lbd*/*lbd *^yolk sac. By contrast, vascular disorganization was milder in *Notch1*^12*f*/*lbd *^compared to *Notch1*^*lbd*/*lbd *^embryos. The expression of Notch1 target genes was low in *Notch1*^12*f*/*lbd *^yolk sac and embryo head, whereas Vegf and Vegfr2 transcripts were increased. The severity of the compound heterozygous *Notch1*^12*f*/*lbd *^yolk sac phenotype suggested that the allelic products may functionally interact. By contrast, compound heterozygotes with *Notch1*^12*f *^in combination with a *Notch1 *null allele (*Notch1*^tm1Con^) were capable of surviving to birth.

**Conclusions:**

Notch1 signaling in *Notch1*^12*f*/*lbd *^compound heterozygous embryos is more defective than in compound heterozygotes expressing a hypomorphic *Notch1*^12*f *^allele and a *Notch1 *null allele. The data suggest that the gene products Notch1^lbd ^and Notch1^12f ^interact to reduce the activity of Notch1^12f^.

## Background

Notch transmembrane receptors are important regulators of cell fate determination in numerous cell types [1-3]. Notch receptors in Drosophila and mammals are covalently modified with *O*-fucose on many epidermal growth factor-like (EGF) repeats of the extracellular domain [4]. An important *O*-fucose site resides in epidermal growth factor-like repeat 12 (EGF12) which, together with EGF11, is required for canonical Notch ligand binding to Drosophila Notch [5-7] and to mammalian Notch1 [8]. A point mutation that precludes the addition of fucose to EGF12 in Drosophila Notch results in enhanced binding of both Delta and Serrate Notch ligands, and a hyperactive Notch that is refractory to Fringe [9]. However, the same mutation (*Notch1*^12*f*^) in cultured mammalian cells gives markedly reduced signaling in a Notch reporter signaling assay [10,11], predicting a Notch1 null phenotype *in vivo*. Surprisingly however, homozygous *Notch1*^12*f*/12*f *^mice are viable and fertile, but exhibit retarded growth and mild defects in T cell development in the thymus [12], consistent with weak Notch1 signaling. *Notch1*^+/12*f *^heterozygotes are indistinguishable from wild type in terms of growth and T cell development. However, compound heterozygotes carrying *Notch1*^12*f *^and the inactive *Notch1*^*lbd *^allele, which lacks the ligand binding domain and generates an inactive ~280 kDa Notch1 receptor at the cell surface, are not born [12]. Therefore *Notch1*^12*f *^is a hypomorphic allele in mammals and the *O*-fucose glycan in the ligand binding domain is required for optimal Notch1 signaling. Homozygous *Notch1*^*lbd*/*lbd *^embryos die at ~E9.5 [8,12] with an indistinguishable phenotype compared to Notch1 null embryos (*Notch1*^*in*32/*in*32 ^and *Notch1*^*tm*1*Con*/*tm*1*Con*^) described by others [13,14]. Heterozygous *Notch1*^+/*lbd *^and *Notch1*^+/*tm*1*Con *^mice are viable and fertile whereas *Notch1*^12*f*/*lbd *^compound heterozygotes die between E11.5 and E12.5, significantly later than either *Notch1*^*lbd*/*lbd *^[12] or *Notch1 *null embryos [13,14] that do not express Notch1 [15-17].

The availability of these *Notch1 *mutant alleles suggested a genetic approach to determining effects of varying Notch1 signaling strength. The *Notch1*^*lbd *^mutation generates a non-functional but cell surface-expressed Notch1 that cannot signal [8,12]. *Notch1*^*tm*1*Con *^lacks Notch1 on the cell surface due to the absence of its transmembrane domain [14]. *Notch1*^*in*32 ^homozygous embryos have no Notch1 transcripts [13] and an indistinguishable phenotype from *Notch1*^*tm*1*Con *^homozygotes which lack Notch1 based on western analyses [15,18]. *Notch1*^+/- ^heterozygotes carrying either of the *Notch1 *null alleles exhibit Notch1 signaling defects in certain cell types, an effect attributed to Notch1 haploinsufficiency rather than to a dominant negative effect in *Notch1*^*tm*1*Con *^[18-21]. In this paper we compare embryogenesis and vasculogenesis in compound heterozygotes expressing the hypomorphic *Notch1*^12*f *^allele with either the inactive *Notch1*^*lbd *^allele [8,12] or the *Notch1*^*tm*1*Con *^null allele [14].

## Results

### Notch signaling in Notch1^12f/lbd ^compound heterozygous ES cells

**Figure 1:**
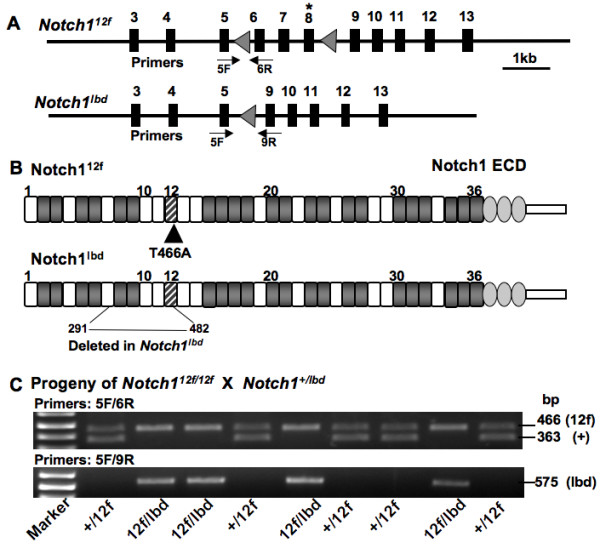
**Generation of *Notch1*^12*f*/*lbd *^embryos**. (A) Diagram of the *Notch1*^12*f *^and *Notch1*^*lbd *^alleles. (B) Diagram of mouse Notch1 EGF repeats in Notch1^12*f *^and Notch1^lbd ^extracellular domains. The EGF repeats with putative *O*-fucosylation sites are shaded in gray and the mutation in EGF12 is shown. (C) Genotyping by PCR from E9.5 yolk sac DNA of a litter from a *Notch1*^12*f*/12*f *^× *Notch1*^+/*lbd *^cross. Primers 5F and 6R detect the *Notch1*^12*f *^and *Notch1*^+ ^alleles, primers 5F and 9R detect the *Notch1*^*lbd *^allele.

**Figure 2:**
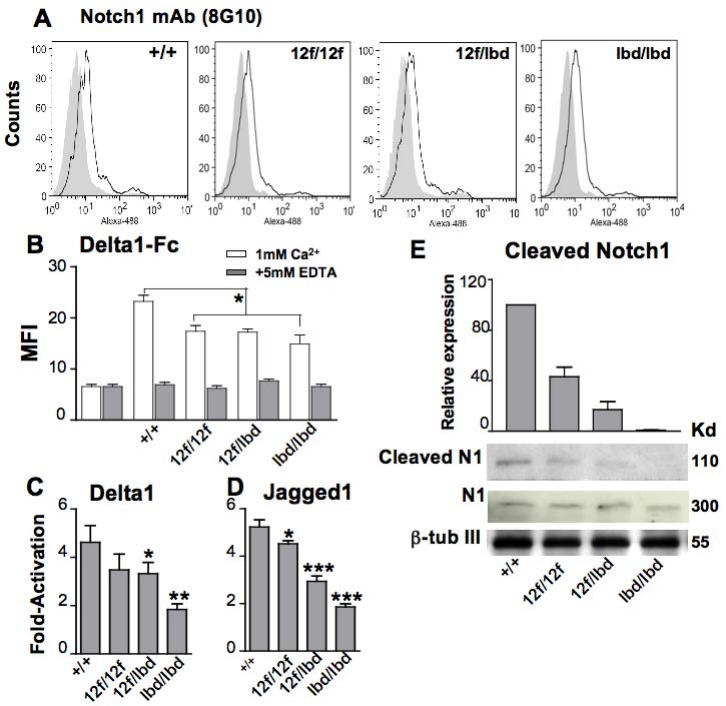
**A graded reduction in Notch1 signaling in *Notch1*^12*f*/*lbd *^ES cells**. (A) Notch1 expression on the surface of ES cells (*Notch1*^+/+^, *Notch1*^12*f*/12*f*^, *Notch1*^12*f*/*lbd *^and *Notch1*^*lbd*/*lbd*^) was analyzed by flow cytometry using anti-Notch1 mAb 8G10 (solid line). Shaded profiles are secondary Ab only. (B) Delta1-Fc binding to ES cells. Control is secondary antibody alone. 5 mM EDTA inhibited ligand binding to control levels (gray). Data are mean ± SEM (n = 4), * *p *< 0.05 between *Notch1*^+/+ ^and all mutant lines. (C) Delta1-induced Notch signaling and (D) Jagged1-induced Notch1 signaling were determined by co-culturing ES cells with Delta1/L or Jagged1/L cells compared to control L cells after transfection of a Notch reporter construct. Bars represent fold-activation ± SEM (n = 4), * *p *< 0.05; ** *p *< 0.01, *** *p *< 0.001 based on the two-tailed Student's t test; (E) Whole cell lysates from ES cells were subjected to western analysis using the Val1744 antibody for activated Notch1 and the 8G10 antibody for full length Notch1. The histogram shows the relative expression of activated Notch1 after normalization to β-tubulin III (mean ± SEM from 4 experiments).

The *Notch1*^12*f *^and *Notch*^*lbd *^alleles investigated here are diagrammed in Fig. 1A and 1B and their identification by PCR genotyping is shown in Fig. 1C. Previous studies showed that *Notch1*^12*f*/*lbd *^compound heterozygotes die by ~E12.5 [12]. To examine Notch ligand binding and the strength of Notch signaling in more detail, ES cells were derived from *Notch1*^12*f*/*lbd *^compound heterozygous blastocysts and compared to ES cells derived from *Notch1*^12*f*/12*f *^and *Notch1*^*lbd*/*lbd *^homozygous blastocysts and wild type ES cells (Fig. 2). All cell lines bound the anti-Notch1 extracellular domain mAb 8G10 equivalently, and therefore expressed the various Notch1 molecules similarly at the cell surface (Fig. 2A). Each mutant line exhibited a decrease in the low level of soluble Delta1 binding observed with wild type ES cells (Fig. 2B). Binding of Delta1 is not reduced to zero even in *Notch1 *null ES cells because of the presence of Notch2, Notch3 and Notch4 [17]. Notch signaling was analysed in co-culture assays with L cells or L cells expressing full length Delta1 or Jagged1 ligand. This reporter assay revealed a graded reduction in Notch signaling with *Notch1*^12*f*/12*f *^>*Notch1*^12*f*/*lbd *^>*Notch1*^*lbd*/*lbd *^ES cells (Fig. 2C-D). This graded response was also observed by western analysis using Notch1 antibody Val1744 [15] which detects the ~110 kDa Notch1 fragment generated by γ-secretase complex cleavage of Notch1. The level of activated Notch1 in *Notch1*^12*f*/*lbd *^ES cells was less than in *Notch1*^12*f*/12*f *^ES cells, which was lower than in control ES cells, while *Notch1*^*lbd*/*lbd *^ES cells had undetectable levels of activated Notch1 (Fig. 2E). Nevertheless, all ES cell populations, including *Notch1*^*lbd*/*lbd *^ES cells, expressed equivalent levels of full-length Notch1 (Fig. 2E). Taken together, these data indicate that Notch1^12f ^and Notch1^lbd ^expression and transit to the cell surface were similar to wild type Notch1, but Notch1 signaling was reduced in mutant cells: Notch1^12f ^signaling was sightly less than wild type; signaling from the combination of Notch1^12f ^and Notch1^lbd ^was further reduced, and signaling by Notch1^lbd ^alone was essentially absent. Previous experiments have shown that *Notch1*^*lbd*/*lbd *^and *Notch1*^*in*32/*in*32 ^ES cells which lack Notch1 [13,15,16], are equally deficient in Delta1-Fc binding and Notch1 signaling [12].

### Embryogenesis in Notch1^12f/lbd ^compound heterozygous embryos

**Figure 3:**
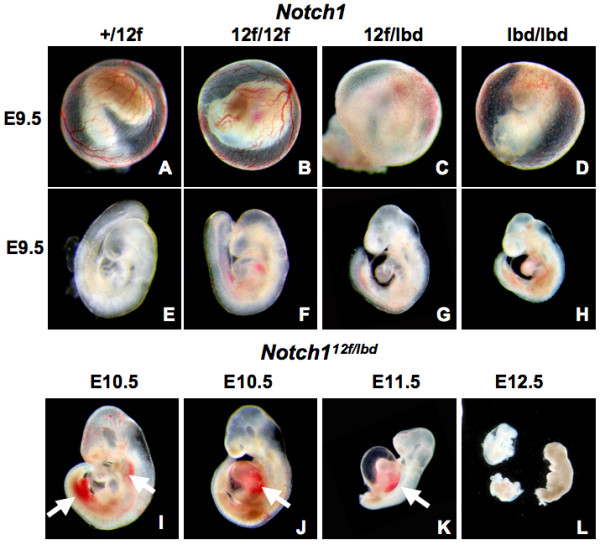
**Embryogenesis in *Notch1*^12*f*/*lbd *^embryos**. (A-D) Vascularization of yolk sac in *Notch1*^+/12*f*^, *Notch1*^12*f*/12*f*^, *Notch1*^12*f*/*lbd *^and *Notchl*^*lbd*/*lbd *^embryos at E9.5. Large vitelline blood vessels were present in *Notch1*^+/12f ^and *Notch1*^12*f*/12*f *^yolk sacs, but absent in the *Notch1*^12*f*/*lbd *^and *Notch1*^*lbd*/*lbd *^mutants. (E-H) Morphology of embryos at E9.5. *Notch1*^12*f*/12*f *^are similiar to *Notch1*^+/12*f*^, *Notch1*^12*f*/*lbd *^are markedly underdeveloped, and *Notch1*^*lbd*/*lbd *^are severely underdeveloped. (I-L) *Notch1*^12*f*/*lbd *^embryos from E10.5-E12.5. White arrows show hemorrhaging in E10.5 and E11.5 embryos; most E12.5 embryos were resorbing. The number of embryos examined at each stage is given in Table 1.

Embryonic development was compared between *Notch1*^12*f*/12*f*^, *Notch1*^12*f*/*lbd *^and *Notch1*^*lbd*/*lbd *^embryos. At E9.5 *Notch1*^12*f*/*lbd *^embryos formed 17-21 somites compared to 23-26 somites in *Notch1*^12*f*/12*f *^embryos, the same as *Notch1*^+/+ ^embryos, and 13-17 somites in *Notch1*^*lbd*/*lbd *^embryos [8], the same as *Notch1*^*tm*1*Con *^null embryos [14] (Table 1). Compared to *Notch1*^12*f*/12*f *^and *Notch1*^+/12*f *^embryos, *Notch1*^12*f*/*lbd *^embryos also showed severely defective vasculogenesis in yolk sac at E9.5, similar to *Notch1*^*lbd*/*lbd *^yolk sac. By contrast, *Notch1*^12*f*/*lbd *^embryos at E9.5 and E10.5 exhibited milder defects in development than *Notch1*^*lbd*/*lbd *^embryos [12] (Fig. 3), although the ballooning of the pericardial sac and defective heart development were severe, and similar to mutants globally defective in Notch signaling such as mutants lacking Pofut1 [22], RBPJk [23] or presenilins 1 and 2 [24]. Taken together, these data indicate that two copies of *Notch1*^12*f *^do not noticeably affect mouse embryogenesis at a gross level, whereas a single copy of *Notch1*^12*f *^with *Notch1*^*lbd *^support embryonic development ~2.0-2.5 days longer than embryos with two copies of *Notch1*^*lbd*^.

**Table 1:** Somite Numbers in *Notch1 *Mutants

Genotype	Stage	No. Embryos	No. Somites
+/12f or +/+	E9.5	8	23,23,24,24,24,25,25,26
12f/12f	E9.5	4	23,24,25,26
12f/lbd	E9.5	6	17,17,18,18,19,21
12f/tm1Con	E9.5	7	18,19,21,21,23,24,26
lbd/lbd	E9.5	6	13,14,14,16,17,17,
tm1Con/tm1Con	E9.5	*	≤ 14
			
+/12f or +/+	E10.5	3	33,34,35
12f/12f	E10.5	3	32,32,34
12f/lbd	E10.5	5	18,18,21,22,23

Somites were counted in embryos of *Notch1*^+/+^, *Notch1*^+/12*f*^, *Notch1*^12*f*/12*f*^, *Notch1*^12*f*/*lbd*^, *Notch1*^12*f*/*tm*1*Con *^and *Notch1*^*lbd*/*lbd *^at E9.5. *Somite numbers in *Notch1*^*tm*1*Con*/*tm*1*Con *^embryos are from Conlon et al. [14].

### Vasculogenesis in yolk sac appears to require stronger Notch1 signaling than in the embryo

**Figure 4:**
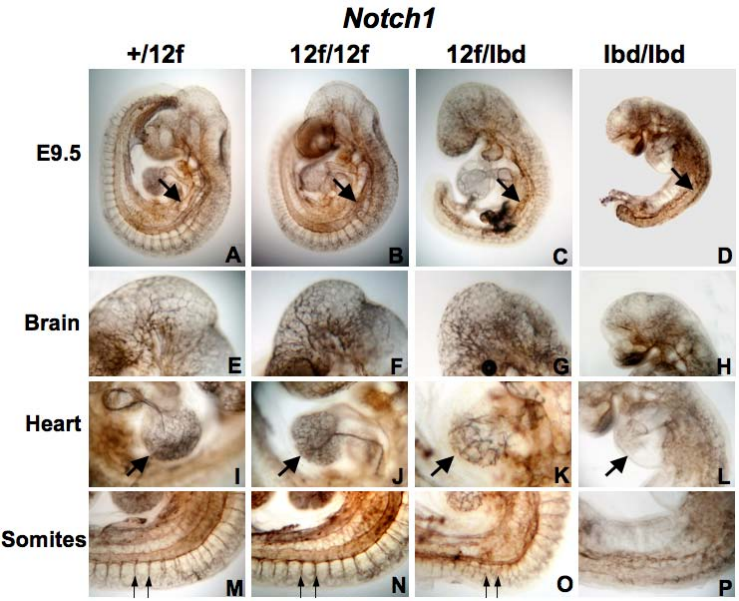
**Defects in vascular remodeling in *Notch1*^12*f*/*lbd *^E9.5 embryos**. All whole mount embryos were stained with Ab to Pecam1. (A-D) Morphogenesis of the main trunk of the anterior cardinal vein (arrow) in *Notch1*^12*f*/*lbd *^and *Notch1*^*lbd*/*lbd *^mutant embryos is defective compared to *Notch1*^12*f*/12*f *^and control *Notch1*^+/12*f *^embryos. (E-H) Vascular remodeling in brain in *Notch1*^+/12*f *^and *Notch1*^12*f*/12*f *^is similar but is defective in *Notch1*^12*f*/*lbd *^and severely defective in *Notch1*^*lbd*/*lbd *^embryos. (I-L) Vascular remodeling in heart is defective in *Notch1*^12*f*/*lbd *^and more severely affected in *Notch1*^*lbd*/*lbd *^embryos. (M-P) Intersomitic vessels (arrows) were well-organized in *Notch1*^+/12*f *^and *Notch1*^12*f*/12*f *^embryos but were mildly disorganized in *Notch1*^12*f*/*lbd *^and essentially absent from *Notch1*^*lbd*/*lbd *^embryos. The number of embryos examined was 3 - 4 of each genotype.

Notch1 signaling is critical for vasculogenesis during mouse embryogenesis [25]. Loss of Notch1 in embryos [26] or in endothelial cells [27] causes embryonic lethality with severe vascularization defects in yolk sac, placenta and embryo. Blood that had leaked from the heart and blood vessels was apparent in *Notch1*^12*f*/*lbd *^embryos (Fig. 3I-K; arrows). Vascular organization in embryos was examined by staining with anti-Pecam1 (endothelial marker platelet/endothelial cell adhesion molecule-1). *Notch1*^12*f*/12*f *^embryos (Fig. 4B, F, J, N) did not exhibit any apparent defects in brain, heart or intersomitic vascularization compared to *Notch1*^+/12*f *^embryos. *Notch1*^12*f*/*lbd *^embryos exhibited somewhat disorganized vascularization in embryos, especially in the main trunk of the anterior cardinal vein, the vascular network of the head and heart, and in intersomitic vessels (Fig. 4C, G, K, O). *Notch1*^*lbd*/*lbd *^embryos exhibited severe defects in vascularization (Fig. 4D, H, L, P). Therefore, the extensive vascularization in E9.5 and older *Notch1*^12*f*/*lbd *^embryos appears to be well supported by the level of Notch1 signaling provided by the *Notch1*^12*f *^allele. Considering that the vascular defects in yolk sac of compound heterozygous *Notch1*^12*f*/*lbd *^and homozygous *Notch1*^*lbd*/*lbd *^embryos were similarly severe, the comparatively milder defects in *Notch1*^12*f*/*lbd *^embryos indicated that Notch1 signaling from a single copy of *Notch1*^12*f*^, while not sufficient to support vascularization in yolk sac at E9.5, is able to support a high level of vascularization in E9.5 embryos. It seems that vascularization in yolk sac requires stronger Notch1 signaling than in the embryo.

### Notch1 target gene expression in E9.5 yolk sac versus embryo

**Figure 5:**
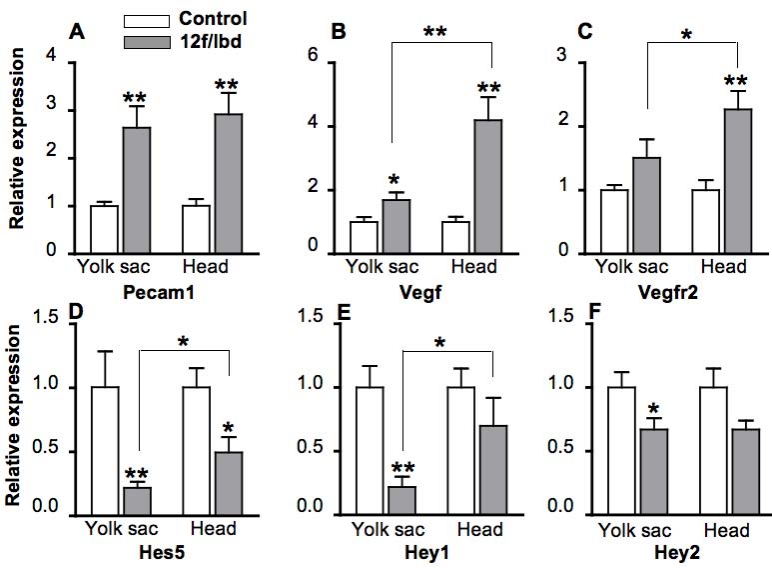
**Real-time PCR of vasculogenic and Notch target genes in *Notch1*^12*f*/*lbd *^yolk sac and embryo**. Total RNA extracted from E10.5 yolk sac or embryonic head was reverse-transcibed and subjected to real-time PCR. Numbers of transcripts were normalized to β-actin, and the average relative expression of *Notch1*^+/12*f *^samples was set to 1. (A-F) Relative expression of *Pecam1*, *Vegf*, *Vegfr2*, *Hes5*, *Hey1*, and *Hey2 *as marked. Bars represent SEM (n = 6). The two-tailed Student's t test was used in control versus mutant yolk sac and embryo head comparisons; a one-tailed Student's t test was used in mutant yolk sac versus mutant embryo head comparisons; * *p *< 0.05; ** *p *< 0.01

**Figure 6:**
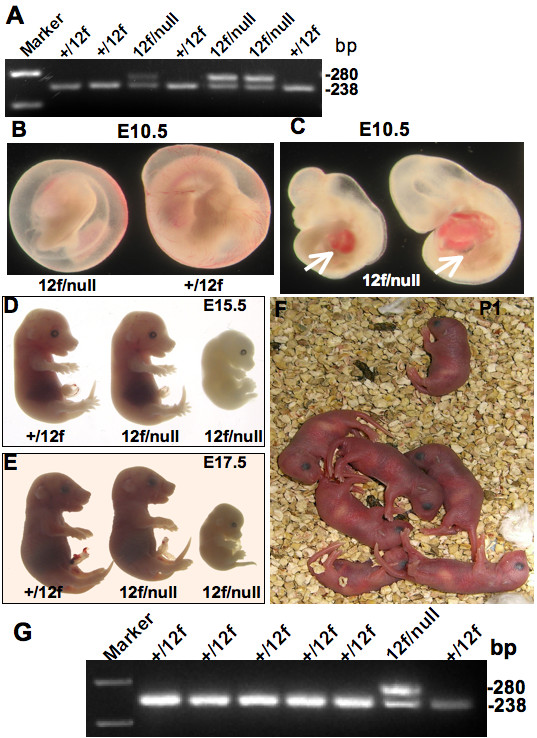
***Notch1*^12*f*/*tm*1*Con *^embryos survive longer than *Notch1*^12*f*/*lbd *^embryos**. (A) PCR genotype of an E9.5 litter showed the 280 bp PCR product from *Notch1*^*tm*1*Con *^allele and the 238 bp product from the *Notch1*^12*f *^allele. (B) Yolk sac vascularization of E10.5 *Notch1*^12*f*/*tm*1*Con *^and *Notch1*^+/12*f *^embryos. (C) *Notch1*^12*f*/*tm*1*Con *^embryos at E10.5 exhibit heamorrhaging around the heart (arrows). (D) *Notch1*^12*f*//*tm*1*Con *^and control embryos at E15.5. One *Notch1*^12*f*/*tm*1*Con *^embryo was defective but the other had no obvious defects. (E) *Notch1*^12*f*/*tm*1*Con *^and control embryos at E17.5. One *Notch1*^12*f*/*tm*1*Con *^embryo was defective but the other had no obvious defects. (F) Photo of a litter on postnatal day 1 (P1) which included one pup identified as *Notch1*^12*f*/*tm*1*Con *^by PCR genotyping below. The pup was indistingishable but died within a few days. (G) PCR genotype of the P1 litter in panel F.

Whereas vascularization was severly affected in both yolk sac and embryo of *Notch1*^*lbd*/*lbd *^embryos, only the yolk sac of *Notch1*^12*f*/*lbd *^compound heterozygous embryos exhibited extremely defective vascularization. To investigate further, the expression of vasculogensis-related and Notch1 target genes was examined by real-time PCR using total RNA isolated from E10.5 *Notch1*^12*f*/*lbd *^and *Notch1*^+/12*f *^yolk sacs and embryo heads. The relative expression levels of Pecam1 and Vegf were increased in *Notch1*^12*f*/*lbd *^yolk sacs and embryos, and Vegfr2 expression was increased in *Notch1*^12*f*/*lbd *^embryo heads (Fig. 5A-C). Therefore loss of Notch1 signaling upregulated transcription of the *Pecam1, Vegf and Vegfr2 *genes. Interestingly, the increased expression of *Vegf *and *Vegfr2 *was greater in *Notch1*^12*f*/*lbd *^embryos, consistent with the relative strength of Notch1 signaling being greater in yolk sac. Expression of the Notch1 target genes *Hes5, Hey1 *and *Hey2 *was reduced in *Notch1*^12*f*/*lbd *^yolk sac (Fig. 5D-F), but the level of *Hes1 *transcripts was not changed (data not shown). In embryos, only the expression of *Hes5 *was significantly reduced compared to control. The expression of *Ang1, Tie2 *and *Ephrin-B2 *which are involved in angiogenesis, as well the expression of *Notch1 *itself, were not changed in *Notch1*^12*f*/*lbd *^yolk sac or embryos (data not shown). The fact that the increase in *Vegf *and *Vegfr2 *transcripts was more in embryo head than yolk sac (418% vs 170% for *Vegf*; 227% vs. 148% for *Vegfr2; *Fig. 5B and 6C), and the fact that the reduction in Notch target gene expression was greater in yolk sac than embryo head, correlated generally with Notch1 signal strength and the greater severity of vascularization defects in yolk sac versus embryo head.

### Notch1^12f ^may function to birth in the absence of Notch1^lbd^

The severity of the *Notch1*^12*f*/*lbd *^phenotype suggested an interaction between Notch1^12f ^and Notch1^lbd ^that interfered with signaling by Notch1^12f^. In this case, compound heterozygous embryos expressing a *Notch1*^12*f *^allele and a Notch1 null allele might be expected to have a milder phenotype than *Notch1*^12*f*/*lbd *^embryos. *Notch1*^12*f*/12*f *^mice were crossed with *Notch1*^+/*tm*1*Con *^heterozygotes and embryos were examined at E9.5 and later (Fig. 6, Table 2). Some *Notch1*^12*f*/*tm*1*Con *^embryos died between E11.5 and E12.5 with similar defects to *Notch1*^12*f*/*lbd *^embryos. However, this is ~1.5 days later than observed with *Notch1*^*tm*1*Con*/*tm*1*Con *^homozygous embryos who were mostly dead by E10 [14]. However, nearly one third of the *Notch1*^12*f*/*tm*1*Con *^embryos developed beyond E12.5 and died at various times during embryogenesis, including after birth (Table 2). Two *Notch1*^12*f*/*tm*1*Con *^pups were found after birth, but none were observed after postnatal day 7 (Fig. 6, Table 2). Somite numbers in *Notch1*^12*f*/*tm*1*Con *^embryos varied from as low as *Notch1*^12*f*/*lbd *^embryos to as high as wild type embryos (Table 1). Taken together, these results indicate that Notch1^12f ^receptors present at a 50% dose *in vivo*, generate stronger Notch1 signaling than Notch1^12f ^in combination with Notch1^lbd^. This provides genetic evidence that Notch1^12f ^and Notch1^lbd ^may functionally interact.

**Table 2:** *Notch1*^12*f*/*tm*1*Con *^pups may survive to birth

Stage	12f/12f × +/tm1Con							12f/12f × +/lbd			
	**Litters**	**Pups**	**+/12f**	**12f/tm1Con**				**Litters**	**Pups**	**+/12f**	**12f/lbd**
					
E9.5	3	23	11	12				5	38	18	20
E10.5	3	23	13	10				8	70	37	33
E11.5	4	24	16	8				7	48	28	20
E12.5	5	25	21	4				8	33	28	5
E13.5	5	27	21	6				5	23	23	0
E15.5	3	13	9	4				-	-	-	-
E17.5	5	19	17	2				-	-	-	-
P1	6	25	23	2				-	-	-	-
Wean	13	50	50	0				8	30	30	0

Embryos (E9.5 -- E17.5), post-natal pups (P1) and mice after weaning were genotyped from crosses of *Notch1*^12*f*/12*f *^female × *Notch1*^+/*tm*1*Con *^male or crosses of *Notch1*^12*f*/12*f *^female × *Notch1*^+/*lbd *^male. The data from *Notch1*^+/12*f *^× *Notch1*^12*f*/*lbd *^crosses were previously published [12] but are included for ease of comparison.

## Discussion and Conclusions

In this paper we show that Notch1 signaling is greatly reduced in *Notch1*^12*f*/*lbd *^ES cells and compound heterozygous embryos, but is significantly greater than in *Notch1*^*lbd*/*lbd *^ES cells or homozygous embryos. The presence of the hypomorphic *Notch1*^12*f *^allele allows vasculogenesis to proceed further and embryos to survive ~1.5-2 days longer. The vascular system develops early during mammalian embryogenesis. Initially, endothelial cell precursors differentiate and coalesce into a primitive network of undifferentiated blood vessels (the primary vascular plexus) in both the mammalian embryo and its extraembryonic membrane the yolk sac, in a process termed vasculogenesis [28]. Subsequently, the primary vascular plexus is remodeled into a highly organized and functionally competent vascular network in a process termed angiogenesis [29,30]. These processes are controlled by several signaling molecules, including vascular endothelial growth factor (VEGF) and its receptors [31], angiopoeitin 1 and its receptor Tie2 [32], Ephrin-B ligands and EphB receptors [33], TGFβ and its receptors [34], and Notch receptors and their ligands Delta and Jagged [25,26,35-38]. Defects in vasculogenesis are one of the major reasons that Notch1 null embryos die at mid-gestation [13,26]. Conditional mutation of *Notch1 *in vascular endothelial cells using the *Tie2-Cre *transgene showed that embryos lacking endothelial cell *Notch1 *die at ~E10.5 with profound vascular defects in placenta, yolk sac, and the embryo proper [27]. The Notch1 target genes *Hey1 *and *Hey2 *are also essential for embryonic vascular development [39]. A requirement for Notch signaling in the maintenance of vascular homestasis and the repression of endothelial cell proliferation is also indicated in adult mice by conditional deletion of RBP-Jκ in endothelial cells [40].

Interestingly, *Notch1*^12*f*/*lbd *^embryos allowed us to observe that vasculogenesis is regulated to different extents in yolk sac and embryo by Notch1 signaling. Thus, vascular defects in *Notch1*^12*f*/*lbd *^yolk sac were as severe as in *Notch1*^*lbd*/*lbd *^yolk sac, but vascular defects in *Notch1*^12*f*/*lbd *^embryo heads were comparatively mild. The vasculogenic phenotype of *Notch1*^12*f*/*lbd *^embryos was also milder than reported for *Jagged1 or Notch1 or Notch1/4 *null embryos [13,26,37], reflecting the presence of a low level of Notch1 signaling in *Notch1*^12*f*/*lbd *^compound heterozygotes. The reduced strength of Notch1 signaling was responsible for defective artery development in Delta-like 4 (*Dll4*) heterozygous embryos [38]. *Hes5 *and *Hey1 *are Notch1 target genes, and Notch1 downregulates expression of *Hesr-1*/*Hey1 *thereby enhancing expression of its target gene *Vegfr2 *in endothial cells [41]. In addition, *Vegf *is upregulated six-fold in *Hey1/2 *double knock-out embryos [39]. Notch1 has also been proposed to regulate vasculogenesis and angiogenesis via induction of *Ephrin-B2 *[42,43] and *Ang1 *[44,45], and suppression of *Vegfr-2/Kdr *[41,46]. Consistent with this, we observed enhanced suppression of *Vegfr2 *and *Vegf *in *Notch1*^12*f*/*lbd *^yolk sac and embryo. However, we observed no change in the expression of *Ang1*, *Tie2*, *Ephrin-B2 *or *Notch1 *itself, although experiments in human endothelial cells indicate that *Ang1 *and *Tie2 *are Notch1 target genes [44,45]. Ephrin-B2 was reported to respond to Notch4, but not to Notch1, through Delta-like 4 in differentiating HUVEC cells [43], so it was perhaps not surprising that Ephrin-B2 expression was unchanged in *Notch1*^12*f*/*lbd *^yolk sac or embryo. Thus, decreased Notch1 signaling may inhibit vascular development in yolk sac more than in embryos by inducing more *Vegf *and *Vegfr2 *through generating less *Hes5 *and *Hey1 *mRNA in yolk sac.

The prolonged embryonic development supported by the hypomorphic *Notch1*^12*f *^allele was only ~1.5-2 days for *Notch1*^12*f*/*lbd *^embryos compared to *Notch1*^*lbd*/*lbd *^[8,12], or *Notch1 *null embryos [13,14]. By contrast *Notch1*^12*f*/12*f*^, *Notch1*^+/*lbd *^or *Notch1*^+/- ^heterozygotes are viable and fertile [12-14,20]. This suggests that *Notch1*^*lbd *^may interfere with *Notch1*^12*f *^in a process termed negative complementation for *Abruptex *Notch mutants in Drosophila [47,48]. The basis of negative complementation is most commonly attributed to the products of the mutant alleles interacting physically [47]. Thus Notch1^lbd ^may either be dominant negative and inhibit Notch1^12f ^activity, or may not form a functional dimer or higher oligomer with Notch1^12f^, if that is required for Notch1 to function. We prefer the latter hypothesis because there is no evidence to date that Notch1^lbd ^behaves as a dominant negative in *Notch1*^+/*lbd *^heterozygotes [8,12]. Unfortunately, attempts to prove the existence of dimers or higher oligomers of Notch1 expressed at endogenous levels have so far been unsuccessful and previous attempts came to opposite conclusions. While two groups found that overexpressed Notch1 transfected into cultured cells may form dimers through the transmembrane domain or the extracellular domain EGF repeats, one group concluded that dimerization is necessary for Notch1 to signal [49], while the other concluded that Notch1 signals without the need for dimerization, and is present mainly as a monomer on the cell surface [50]. Both studies characterized transiently-transfected Notch1 expressed at much higher levels than endogenous Notch1, which might induce anomolous interactions.

**Figure 7:**
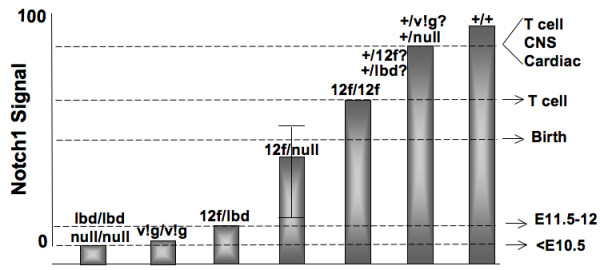
**An allelic series of *Notch1 *mutants**. Based on data reported herein and from the literature, the relative signaling strength of *Notch1 *mutant alleles in various combinations with wild type or other *Notch1 *mutant alleles is represented as discussed in the Discussion. The consequences with respect to time of death of embryos with severe Notch1 signaling defects, or more subtle defects in T cell, CNS or cardiac development are noted.

If Notch1^lbd ^reduces the effective amount of Notch1^12f ^to a level insufficient to sustain development, we reasoned that Notch1^12f ^expressed in the context of a Notch1 null background may function better. In fact, we found that a significant proportion of *Notch1*^12*f*/*tm*1*Con *^embryos survived beyond E12.5 and that some survived to birth. On the other hand, some compound heterozygous *Notch1*^12*f*/*tm*1*Con *^embryos died at ~E11.5 with similar defects to *Notch1*^12*f*/*lbd*^. This indicates that Notch1^12*f *^at a dose of 50% functions at a threshold of Notch1 signaling strength that variably sustains embryogenesis through to birth - a stochastic effect or perhaps a genetic background effect, since *Notch1*^+/12*f *^and *Notch1*^+/*lbd *^mice were not extensively backcrossed to C57Bl/6. Nevertheless, the Notch1 signal strength generated by a single copy of Notch1^12f ^was intermediate between *Notch1*^12*f*/12*f *^and *Notch1*^12*f*/*lbd*^, revealing the importance of maintaining a certain level of Notch1 signaling for mouse embryogenesis to proceed. Fig. 7 summarizes these findings in a diagram which describes a mini-allelic series of available Notch1 mutants. It includes the *Notch1 *processing point mutant Val1744Gly (*Notch1*^*v!g*/*v!g*^) which has a phenotype very similar to, but slightly less penetrant than, a *Notch1 *null [15]. It also includes *Notch1*^+/*null *^heterozygotes that have mild Notch1 signaling defects uncovered in competition assays [19] or by close examination of specific cell types [18,20,21]. Haploinsufficiency of *NOTCH1 *is the basis of aortic valve disease in humans [51]. We predict that *Notch1*^+/*lbd *^and *Notch1*^+/12*f *^heterozygotes have slightly less Notch1 signaling than *Notch1*^+/*tm*1*Con *^and should display evidence of more extensive Notch1 signaling defects in particular cell types. The range of *Notch1 *mutant alleles available in the mouse should be helpful in identifying new *in vivo *functions for Notch1.

## Methods

### Mice

Mice carrying Notch1 lacking the *O*-fucose site in EGF12 (*Notch1*^12*f*^) and mice carrying Notch1 lacking the ligand binding domain (*Notch1*^*lbd*^) were generated by gene targeting as previously described [8,12]. They were backcrossed 6-7 generations to C57/Bl6 mice before being used in these experiments. *Notch1*^*l*2*f*/*lbd *^embryos were obtained by crossing *Notch1*^12*f*/12*f *^and *Notch1*^+/*lbd *^mice. Embryos were collected from E9.5 and yolk sac DNA was genotyped by PCR using primers 5F: GTATGTATATGGGACTTGTAGGCAG and 6R: CTATGAGGGGTCACAGGACCAT, that give a 466 bp product for the *Notch1*^12*f *^allele and a 363 bp product for the wild type *Notch1 *allele; and primers 5F and 9R: CTTCATAACCTGTGGACGGGAG that give a 575 bp product for the *Notch1*^*lbd *^allele. The *Notch1 *null allele (*Notch1*^*tm*1*Con*^) encoding Notch1 lacking the transmembrane domain [14] backcrossed extensively to C57Bl/6 was kindly provided by Cynthia Guidos, University of Toronto. *Notch1*^12*f*/*tm*1*Con *^embryos were obtained by crossing *Notch1*^12*f*/12*f *^and *Notch1*^+/*tm*1*Con *^mice and genotyped by PCR using primers neo-F: CTTGGGTGGAGAGGCTATTC and neo-R: AGGTGAGATGACAGGAGATC for the *Notch1*^*tm*1*Con *^allele and primers loxF: GGCGAGCTCGAATTGATCC and 9R for *Notch1*^12*f *^allele. Mice were housed under conventional barrier protection in accordance with Einstein and NIH guidelines. Protocols were approved by the Albert Einstein Animal Institute Committee.

### Embryonic stem cell isolation

ES cells were isolated from E3.5 blastocysts as described [52], and genomic DNA was genotyped by PCR as described above. ES cells were routinely cultured on an SNL2 γ-irradiated feeder layer [53] in DMEM supplemented with 15% fetal bovine serum (Gemini, West Sacramento, CA), non-essential amino acids, L-glutamine, 1000 U ESGRO^® ^(Chemicon, Temecula, CA), 1% β-mercaptoethanol, 25 mM HEPES, penicillin (50 U/ml) and streptomycin (50 μg/ml). All reagents were from SpecialtyMedia, Lavellette, NJ. Before use in experiments, ES cells were passaged on gelatinized plates for 2-3 generations to remove feeder cells.

### Western blot analysis

ES cells cultured on gelatinized plates were lysed in RIPA buffer (Upstate, Lake Placid, NY) containing complete protease inhibitor cocktail (Roche, Basel, Switzerland) for 30 min on ice and debris was removed by low speed centrifugation. Lysates were resolved by SDS-PAGE, transferred to polyvinyldifluoride (PVDF) membrane and probed with 8G10 anti-Notch1 mAb (Upstate, 57-557, 1:500, Lake Placid, NY) for full-length Notch1 or Val1744 Notch1 antibody (Cell Signaling Technology, Val1744, 1:1000, Beverly, MA) for cleaved, activated Notch1, followed by horseradish peroxidase(HRP)-conjugated secondary antibodies. Reactive bands were visualized with Enhanced Chemiluminescence Reagent (Amersham Pharmacia Biotech, Piscataway, NJ). β-tubulin-III specific antibody T8660 (Sigma Chemical Co., St. Louis, MO) was used as a loading control.

### Flow cytometry

For cell surface Notch1 expression, 70-80% confluent ES cells were dissociated from plates using phosphate-buffered saline (PBS)-based enzyme-free dissociation solution (SpecialtyMedia, Lavellette, NJ) for 10 min at 37°C. After washing, ES cells (5 × 10^5^) were incubated with 0.5 μg 8G10 anti-Notch1 antibody in Hank's balanced salt solution containing 3% bovine serum albumin Fraction V (Sigma Chemical Co., St. Louis, MO), 1 mM CaCl_2 _and 0.05% Na azide (HBSS/BSA) for 1 h at 4°C, washed and incubated in Alexa-488 conjugated anti-Hamster IgG (1:100) in HBSS/BSA in the dark (Invitrogen, Carlsbad, CA) for 30 min at 4°C. Immunofluorescence was analyzed on a FACSCalibur flow cytometer (BD Biosciences, San Diego, CA), gating on live cells determined by 7-AAD staining. Data were analyzed using Flowjo software (Tree Star, San Carlos, CA).

### Notch co-culture signaling assay

Notch signaling assays were performed in duplicate as previously described [54,55]. ES cells were plated at 2 × 10^5 ^cells per well of a six-well plate in ES medium, and co-transfected the next day with 0.2 μg of TP1-luciferase Notch reporter plasmid and 0.05 μg of *Renilla *luciferase reporter (pRL-TK; Promega, Madison, WI) along with 1.8 μg empty vector alone using FuGene 6 (Roche, Basel, Switzerland). At 16 h post-transfection, ES cells were overlaid with 1 × 10^6 ^rat Jagged1-expressing L cells (Jagged1/L), Delta1-expressing L cells (Delta1/L) or parental L cells [56]. At 48 h after transfection, firefly and *Renilla *luciferase activities were quantitated in cell lysates using a dual luciferase assay (Promega, Madison, WI). Ligand-dependent Notch activation was expressed as relative fold-activation of normalized luciferase activity stimulated by ligand/L cells compared to L cells.

### Notch ligand binding assay

Soluble Notch ligand Delta1 with human Fc tag [57,58] was prepared form HEK-293T cells expressing Delta1-Fc [17] cultured in α-MEM containing 10% FBS until 70~80% confluence. The medium was changed to 293 SFM II serum-free medium (Invitrogen) and conditioned medium was collected after 3 days. Cellular debris was removed by low-speed centrifugation, the supernatant was filtered and stored at 4°C. Soluble ligand concentration was determined by western blotting using HRP-conjugated anti-human IgG antibody (Jackson Immunoresearch, West Grove, PA). For the binding assay, ES cells on plates were dissociated using PBS-based Enzyme-free dissociation medium for 10 min at 37°C, and the single cell suspension of ES was incubated with 2 μg/ml Delta1-Fc in HBSS/BSA for 1 h at 4°C, followed by incubation with 1:100 phycoerythrin (PE)-conjugated anti-human Fc antibody (Jackson Immunoresearch, West Grove, PA) for 30 min at 4°C. After washing, live cells determined by gating on the 7-AAD negative population were analyzed on a FACS Calibur flow cytometer (BD Biosciences, San Jose, CA). Ligand binding ability was measured as mean fluorescence intensity (MFI) using Flowjo software (Tree Star, San Carlos, CA).

### Whole mount immunohistochemistry

Embryos were collected on E9.5 and DNA from yolk sac was genotyped by PCR. Embryos were fixed in 4% paraformaldehyde (PFA) in PBS overnight at 4°C, dehydrated through a methanol series, and bleached in 5% H_2_O_2_/methanol for 5 h. Embryos were rehydrated and placed in PBSMT (PBS containing 3% nonfat milk, 0.1% Triton X-100). After 2 h, embryos were incubated with anti-mouse Pecam1 (1:200; BD Biosciences, San Jose, CA) in PBSMT overnight at 4°C. After 5 washes with PBSMT embryos were incubated in a 1:200 dilution of HRP-conjugated secondary antibody (Zymed, South San Francisco, CA) overnight. Embryos were washed 5 times in PBSMT and rinsed in PBT (PBS containing containing 0.2% BSA, 0.1% Triton X-100), followed developing with DAB kit (Vector Laboratories, Burlingame, CA). Finally, embryos were washed in PBT and postfixed in 4% PFA, dehydrated through a methanol series and cleared in BABB (benzyl alcohol: benzyl benzoate - 1:2) in a glass Petri dish. Photos were taken in PBS or BABB using an inverted phase contrast microscope (Olympus IMT-2, Olympus America Inc., Center Valley, PA) and a Canon S40 camera with T-mount adaptor.

### Real-Time PCR

Total RNA was extracted from yolk sac or embryo head using TRIZOL^® ^reagent (Invitrogen, Carlsbad, CA) according to the manufacturer's instructions. Aliquots of 1 μg RNA were digested by DNase I and cDNA was prepared using RNA PCR Kit ver. 3.0 (Takara Mirus Bio, Madison, WI) with oligo dT priming. Real-time PCR reactions with SybrGreen quantification were established with 1/20 of each cDNA preparation in an Opticon2 DNA Engine (MJ Research, Cambridge, MA). Relative expression levels after normalization using β-actin were calculated using the 2^-ΔΔCT ^method ()([59] and confirmed by the absolute quantification method using standard curves. Primer pairs for real-time PCR were *Ang1 *(CATTCTTCGCTGCCATTCTG, GCACATTGCCCATGTTGAATC)[60], *Pecam1 *(GAGCCCAATCACGTTTCAGTTT, TCCTTCCTGCTTCTTGCTAGCT) [60], *Vegf *(GGAGATCCTTCGAGGAGCACTT, GCGATTTAGCAGCAGATATAAGAA)[60], *Tie2 *(ATGTGGAAGTCGAGAGGCGAT, CGAATAGCCATCCACTATTGTCC)[60], *Hey1 *(TGAGCTGAGAAGGCTGGTAC, ACCCCAAACTCCGATAGTCC)[39], *Hey2 *(TGAGAAGACTAGTGCCAACAGC, TGGGCATCAAAGTAGCCTTTA)[39], *Ephrin-B2 *(GCGGGATCCAGGAGATCCCCACTTGGACT, GTGCGCAACCTTCTCCTAAG)[39], *Hes1 *(AAGGCGGACATTCTGGAAAT, GTCACCTCGTTCATGCACTC) [61]. *Hes5 *(TACCTGAAACACAGCAAAGC, GCTGGAGTGGTAAGCAG) [62] and β-actin (GTGGGCCGCTCTAGGCACCA, TGGCCTTAGGGTTCAGGGGG). All real-time PCR experiments were performed in duplicate from ≥ 4 independent samples.

### Statistical analysis

Statistical significance was calculated using the unpaired Student's *t*-test (two-tailed) using Graphpad Prism (GraphPad Software, Inc., San Diego, CA) unless otherwise noted.

## Authors' contributions

PS conceived the project, obtained funding, participated in the design of experiments and analysis of data, and co-wrote the manuscript; CG partipated in the design of the experiments, performed or participated in all experiments, analysed data and co-wrote the paper. All authors read and approved the final version of the manuscript.
